# The prevalence and clinical relevance of *2R/2R TYMS* genotype in patients with gastrointestinal malignancies treated with fluoropyrimidine-based chemotherapy regimens

**DOI:** 10.1038/s41397-021-00210-2

**Published:** 2021-02-19

**Authors:** Moh’d Khushman, Girijesh Kumar Patel, Anu Singh Maharjan, Gwendolyn A. McMillin, Cindy Nelson, Peter Hosein, Ajay P. Singh

**Affiliations:** 1 Medical Oncology, Mitchell Cancer Institute, The University of South Alabama, Mobile, AL USA; 2 Department of Oncological Sciences, Mitchell Cancer Institute, The University of South Alabama, Mobile, AL USA; 3 Department of Cell Biology and Chemistry, Texas Tech University Health Sciences Center, Lubbock, TX USA; 4 ARUP Laboratories, The University of Utah, Salt Lake City, UT USA; 5 Hematology-Oncology, Sylvester Cancer Center, The University of Miami, Miami, FL USA; 6 Department of Pathology, The University of South Alabama, Mobile, AL USA

**Keywords:** Cancer, Drug regulation

## Abstract

**Introduction:**

The prevalence of 2R/2R *TYMS* genotype is variable but estimated to be around 20–30% in Caucasians. The clinical relevance of *TYMS 2R/2R* genotype in predicting severe fluoropyrimidine-related adverse events (FrAE) is controversial. Here, we explored the prevalence and clinical relevance of *2R/2R TYMS* genotype.

**Methods:**

Between 2011 and 2018, 126 patients were genotyped for *TYMS*. FrAEs were graded according to CTCAE version 5.0. Fisher’s exact test was used for statistical analysis.

**Results:**

The prevalence of *TYMS 2R/2R* genotype was 24.6%. Among patients with TYMS genotypes (*N* = 71) that predict decreased TS expression, *2R/2R TYMS* genotype was the most common *TYMS* genotype seen in female (57%) and African American (60%) patients. Among patients with genotypes that predict increased TS expression (*N* = 55), 12 patients had grade 3–4 FrAEs (22%), while among patients with genotypes that predict decreased TS expression (*N* = 71), 30 patients had grade 3–4 FrAEs (42%) (*p* = 0.0219). Compared to patients with genotypes predicting increased TS expression, 17 out of 31 patients (55%) with *TYMS 2R/2R* genotype had grade 3–4 FrAEs (*p* = 0.0039) and 15 out 40 patients (38%) with *TYMS 2R/3RC* and *TYMS 3RC/3RC* genotype had grade 3–4 FrAEs (*p* = 0.1108).

**Conclusion:**

The prevalence of *TYMS 2R/2R* genotype was 24.6%, and it had a unique sex and ethnic distribution. Polymorphism in the promoter region of *TYMS* gene that predicts decreased TS expression due to *2R/2R* variant was associated with grade 3–4 FrAEs. These data suggest that genotyping patients who are not DPD deficient for TYMS might identify patients at risk of severe FrAEs.

## Introduction

Fluoropyrimidines are antimetabolite chemotherapy drugs that are widely used in the treatment of cancer. There are three fluoropyrimidine drugs in clinical use: intravenous 5-fluorouracil (5-FU), oral capecitabine, and oral tegafur. Capecitabine and tegafur are precursors of 5-FU [1, 2]. Fluoropyrimidines are considered the backbone of most chemotherapeutic regimens approved for the treatment of gastrointestinal (GI) malignancies [3]. They also represent treatment options in other malignancies such as breast and head and neck cancer [4, 5].

Among patients treated with 5-FU or capecitabine, approximately 20–25% of patients experience severe (grade 3–4) fluoropyrimidine-related adverse events (FrAEs) [6]. Severe FrAEs lead to patients’ hospitalization and treatment interruption or discontinuation. The inter-individual variation in the occurrence and severity of FrAEs is partly due to genetic factors [7, 8].

Dihydropyrimidine dehydrogenase (DPD) enzyme, encoded by *DPYD* gene, is the rate-limiting enzyme for 5-FU catabolism, eliminating approximately 80% of administered or formed 5-FU [9]. Any variation in DPD activity can result in a cytotoxic accumulation of free 5-FU. The prevalence of DPD deficiency in Caucasians is approximately 3–5% [10, 11]. African Americans, especially women, seem to have a higher prevalence of approximately 4–12% [12]. Genomic analysis of patients with DPD deficiency has identified over 128 mutations and polymorphisms in the *DPYD* gene, but only four high-risk variants (DPYD*2A, DPYD*13, DPYD*9B, and *HapB3*) have been consistently associated with DPD deficiency and FrAEs. Genotyping for *DPYD* helps in identifying patients with DPD deficiency and guide the dosing of fluoropyrimidines. However, genotyping is limited to high-risk variants, and most patients who experience FrAEs are not DPD deficient [13–15].

**Fig. 1:**
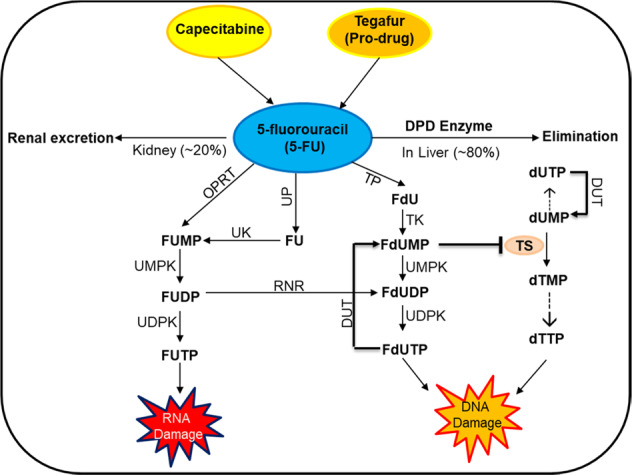
Schematic representation of fluoropyrimidine-based drug metabolic pathway. The capecitabine and tegafur are the oral pre-pro and pro-drug, respectively, which in turn converted into 5-FU, while 5-FU is directly administered as IV. In normal condition of DPD and TS activity, maximum drug is eliminated from body while minimal amount is functionally active and inhibits the DNA and RNA synthesis leading to cell death during cancer treatment. Patients possessing the DPD deficiency show grade 3–4 toxicity as maximum drug is accumulated in the body that inhibit the TYMS. The *TYMS 2R/2R* genotype has low TS level and correlated with severe fluoropyrimidines-related adverse events. DPD dihydropyrimidine dehydrogenase, UP uridine phosphorylase, UK uridine kinase, TK thymidine kinase, TP thymidine phosphorylase, TS thymidylate synthetase, OPRT orotate phosphoribosyltransferase, RNR ribonucleotide reductase, NME1-NME2 nucleoside diphosphate kinase.

In addition to *DPYD*, polymorphism in the *TYMS* gene that encodes thymidylate synthase (TS) may be associated with increased risk of FrAEs. TS is potently inhibited by 5-FU. Cells convert 5-FU to the metabolite fluorodeoxyuridine monophosphate, which binds to TS and inhibits the production of deoxythymidine monophosphate (dTMP). dTMP is essential for DNA replication and repair, so the lack of it leads to cell death [16, 17]. Fig. 1 shows the cascade of metabolic reactions where fluoropyrimidines inhibit TS and eventually lead to DNA damage.

**Fig. 2:**
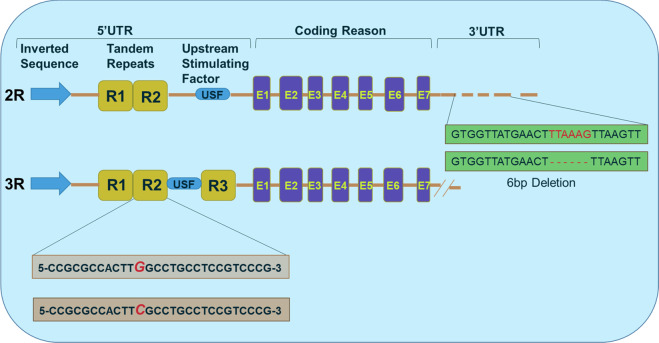
Regulation of TYMS gene expression by 5′ and 3′ untranslated regions (UTRs). Upstream or 5′UTR of the thymidylate synthase gene (TYMS) contains either two tandem repeats (2R) or three tandem repeats (3R) of 28-bp sequences. These tandem repeats regulate the transcription and translation of TYMS gene with the impaired enzyme activity. Moreover, other functional variants of the TYMS gene have been also identified such as single-nucleotide polymorphism (SNP) G>C within the second repeat of the 3R allele. Thymidylate synthase promoter 3RC/3RC genotype causes lower transcriptional activity of TYMS, comparable with the TS *2R/2R* genotype. The six nucleotide insertion or deletion also identified to affect the RNA stability of TYMS gene.

*TYMS* gene expression is regulated by transcription factors that bind to the promoter region. The 5′ untranslated region contains a 28-base-pair variable number of tandem repeats (VNTRs), which act to enhance the promoter and transcriptional activity (Fig. 2). Most patients have either 2 (2R) or 3 (3R) repeats. Homozygous *TYMS 3R/3R* genotype has a higher level of TS, while homozygous *TYMS 2R/2R* genotype has low TS level and may be at greater risk of FrAEs [18, 19]. A single-nucleotide polymorphism of the second repeat of the 3R allele (3RC) abolishes a binding site in the 3R second repeat allele and reduces TS activity compared to wild-type 3R allele (3RG) [20].

The prevalence of 2R/2R *TYMS* genotype in different ethnic background is variable but estimated to be around 20–30% in Caucasians [21]. The clinical relevance of *TYMS 2R/2R* genotype in predicting severe FrAEs is controversial [7, 19, 22–26]. Here, in a cohort of patients with GI malignancies treated with fluoropyrimidine-based chemotherapy regimens, we explored the prevalence and clinical relevance of *2R/2R TYMS* genotype. Moreover, given the different racial and sex background in our cohort, ethnic and sex differences were explored.

## Materials and methods

### Patient population

This is a retrospective study conducted at the University of South Alabama Mitchell Cancer Institute in Mobile, Alabama, USA in collaboration with ARUP Laboratories, The University of Utah, Salt Lake City, Utah, USA. Cohort was identified through searching our cancer center tumor registry for patients with GI malignancies genotyped for *TYMS* gene between 2011 and 2018. The University of South Alabama Institutional Review Board (IRB) approved this study and the IRB-approved database provided a waiver of the requirement for informed consent and allowed for the publication of de-identified data.

### Fluoropyrimidine-based chemotherapy

The fluoropyrimidine-based chemotherapy regimens that the patients in this cohort received include FOLFIRINOX, FOLFOX with or without bevacizumab, cetuximab or panitumumab, XELOX, FOLFIRI with or without bevacizumab, cetuximab or panitumumab, XELIRI with or without bevacizumab, cetuximab or panitumumab, FLOT, 5-FU and mitomycin, 5-FU and liposomal irinotecan, capecitabine and gemcitabine, single agent 5-FU with or without concurrent radiotherapy, and capecitabine with or without concurrent radiotherapy. FOLFIRINOX consists of 5-FU bolus of 400 mg/m^2^, 5-FU continuous infusion 2400 mg/m^2^ for 46 h, leucovorin 400 mg/m^2^, oxaliplatin 85 mg/m^2^, and irinotecan 180 mg/m^2^ every 2 weeks. FOLFOX consists of 5-FU bolus of 400 mg/m^2^, 5-FU continuous infusion 2400 mg/m^2^ for 46 h, leucovorin 400 mg/m^2^, and oxaliplatin 85 mg/m^2^ every 2 weeks. XELOX consists of capecitabine 1000 mg/m^2^ twice daily for 2 weeks and oxaliplatin 130 mg/m^2^ every 3 weeks. FOLFIRI consists of 5-FU bolus of 400 mg/m^2^, 5-FU continuous infusion 2400 mg/m^2^ for 46 h, leucovorin 400 mg/m^2^, and irinotecan 180 mg/m^2^. XELIRI consists of capecitabine 1000 mg/m^2^ twice daily for 2 weeks and irinotecan 250 mg/m^2^ every 3 weeks. FLOT consists of 5-FU continuous infusion 2600 mg/m^2^ for 24 h, leucovorin 400 mg/m^2^, oxaliplatin 85 mg/m^2^, and docetaxel 50 mg/m^2^. 5-FU and mitomycin consist of 5-FU 4000 mg/m^2^ and mitomycin 10 mg/m^2^. 5-FU and nanoliposomal irinotecan consists of 5-FU continuous infusion 2400 mg/m^2^ for 46 h, leucovorin 400 mg/m^2^, and nanoliposomal irinotecan 70 mg/m^2^. Capecitabine and gemcitabine consist of capecitabine 1660 mg/m^2^ for 21 days and gemcitabine 1000 mg/m^2^ day 1, 8, and 15 every 4 weeks.

### Genotyping strategy

Genotyping strategies were quite variable. The most common genotyping strategy was to genotype patients prior to initiating treatment with fluoropyrimidines-based chemotherapy. Between 2016 and 2018, genotyping was conducted almost universally on all patients with GI malignancies treated with fluoropyrimidines-based chemotherapy. Prior to 2016, only 24 patients were genotyped and genotyping was conducted at the discretion of the treating medical oncologist. Those patients were genotyped either because they experienced toxicities or because they had significant comorbidities and their medical oncologist decided to do upfront genotyping.

### *TYMS* genotyping

Germline DNA was obtained from peripheral blood specimens and genotyped *TYMS* gene in ARUP laboratories (Salt Lake City, UT, USA). The VNTRs of the 5’UTR flank of *TYMS* (rs45445694) and their additional single-nucleotide variant (SNV) G>C in the first repeat of the 2R allele (rs183205964, named 2RG or 2RC), the SNV G>C in the second repeat of the 3R allele (rs2863542, named 3RG or 3RC), and the 6 bp insertion in the second repeat of the 3R allele (rs538469385) (all located into the rs45445694 variant) were performed by polymerase chain reaction-restriction fragment length polymorphism. A 6-bp deletion variant at the 3′UTR region of *TYMS* (rs151264360) was genotyped as well.

### *DPYD* genotyping

Germline DNA was obtained from peripheral blood specimens and genotyped for high-risk *DPYD* variants (*IVS14*+*1G*>A [DPYD*2A], DPYD c.1679T>G [DPYD*13A] and *DPYD c.2846A*>T [DPYD*9B]) in ARUP laboratories (Salt Lake City, UT, USA). Only patients with no mutant high-risk *DPYD* variants were included in this cohort. Patients with mutant DPYD*9A (c.85T>*C)* were included in our cohort since the 2017 updated Clinical Pharmacogenetics Implementation Consortium guidelines for DPD genotype and fluoropyrimidine dosing and other studies stated that the DPYD*9A (c.85T>*C)* does not affect DPD activity in a clinically relevant manner [14, 15].

### Toxicity grading and statistical analysis

Demographic and clinical data were extracted from the patients’ charts. FrAEs were graded according to the National Cancer Institute Common Terminology Criteria for Adverse Events (version 5.0). Association between dichotomous fluoropyrimidine-related toxicities and *TYMS* genotype was performed using Fisher’s exact test. Analyses with *p* values ≤ 0.05 were considered significant. Tests were performed using GraphPad software QuickCalcs (GraphPad software 2016, San Diego, CA).

## Results

### Patient characteristics

Between 2011 and 2018, a total of 126 patients with GI malignancies were genotyped for *TYMS* and had no identifiable high-risk *DPYD* variants. The baseline characteristics of the patients are summarized in Table 1. Median age is 59 years. Males represented 55% of the patients, while females represented 45%. In our cohort, 63% were Caucasian, 35% were African Americans, and 2% were other ethnicities (Hispanics, Asians, and Indian Americans). Colon adenocarcinoma represented the most common malignancy in our cohort. Other patients had anal squamous cell carcinoma, appendix adenocarcinoma, cholangiocarcinoma, esophageal adenocarcinoma, gastric adenocarcinoma, neuroendocrine tumor, pancreatic adenocarcinoma, and rectal adenocarcinoma. A fluorouracil-based chemotherapy regimen was administered in 74% of the patients, while 26% of the patients received a capecitabine-based chemotherapy regimen.

**Table 1:** Patient baseline characteristics.

Patient characteristics	Number subject, *N* [%]
Age (years)	
Median (range)	59 (21–90)
Sex	
Female	57 [45%]
Male	69 [55%]
Ethnicity	
African American	44 [35%]
Other ethnicities^a^	3 [2%]
Caucasians	79 [63%]
Diagnosis	
Anal SCC	6 [5%]
Appendix	3 [2%]
Cholangiocarcinoma	4 [3%]
Colon adenocarcinoma	50 [40%]
Esophageal adenocarcinoma	2 [2%]
Gastric adenocarcinoma	8 [6%]
Neuroendocrine tumor (SB)	3 [3%]
Pancreatic adenocarcinoma	13 [10%]
Rectal adenocarcinoma	37 [29%]
Chemotherapy regimen	
Fluorouracil-based	93 [74%]
Capecitabine-based	33 [26%]

*SCC* squamous cell carcinoma, *SB* small bowel.

^a^Hispanics, Asians, and Indian Americans

### *TYMS* genotyping

*TYMS* genotypes that predict increased TS expression *(3RG/3RG, 3RG/3RC, 2R/3RG, 2R/4R, 3R/4R, 4R/3RG)* were identified in 55 patients (44%). *TYMS* genotypes that predict decreased TS expression *(2R/2R, 2R/3RC, 3RC/3RC)* were seen in 71 patients (56%). In our cohort, patients with *2R/2R TYMS* genotype represented 24.6% of the total cohort and 44% of the patients with genotypes that predict decreased expression of TS. *TYMS* genotyping in patients with genotypes that predict increased and decreased TS expression is summarized in Table 2.

**Table 2:** *TYMS* genotyping in patients with GI malignancies with different racial and sex backgrounds.

TYMS genotyping (*N* = 126)	Number of subjects *N* [%]	Sex *N* [%]		Ethnicity *N* [%]		
		Male	Female	Caucasians	AA	Others
Genotypes predictive of increased TS expression	55 [44%]	33 [26.2%]	22 [17.5%]	30 [23.8%]	23 [18.3%]	2 [1.6%]
3RG/3RG	12 [9.5%]	11 [8.7%]	1 [0.8%]	6 [4.8%]	5 [4.0%]	1^a^ [0.8%]
3RG/3RC	22 [17.5%]	10 [7.9%]	12 [9.5%]	19 [15.1%]	3 [2.4%]	0 [0.0%]
2R/3RG	18 [14.3%]	9 [7.1%]	9 [7.1%]	5 [4.0%]	12 [9.5%]	1^b^ [0.8%]
2R/4R	1 [0.8%]	1 [0.8%]	0 [0.0%]	0 [0.0%]	1 [0.8%]	0 [0.0%]
3R/4R	1 [0.8%]	1 [0.8%]	0 [0.0%]	0 [0.0%]	1 [0.8%]	0 [0.0%]
4R/3RG	1 [0.8%]	1 [0.8%]	0 [0.0%]	0 [0.0%]	1 [0.8%]	0 [0.0%]
Genotypes predictive of decreased TS expression	71 [56%]	36 [28.6%]	35 [27.8%]	50 [39.7%]	20 [15.9%]	1 [0.8%]
2R/2R	31 [24.6%]	11 [8.7%]	20 [15.9%]	19 [15.1%]	12 [9.5%]	0 [0.0%]
2R/3RC	31 [24.6%]	19 [15.1%]	12 [9.5%]	24 [19.0%]	7 [5.6%]	0 [0.0%]
3RC/3RC	9 [7.1%]	6 [4.8%]	3 [2.4%]	7 [5.6%]	1 [0.8%]	1^c^ [0.8%]

*AA* African American.

^a^Native American.

^b^Asian.

^c^Hispanic.

### Sex differences

In our cohort, the distribution of *2R/3RG TYMS* genotype was similar between males and females. The distribution of all other genotypes apart from *2R/2R* and *3RG/3RC TYMS* genotypes was more frequent in males than females. The *2R/2R TYMS* genotype had a very unique sex distribution where 20 out of 31 patients (65%) were females. This is the only *TYMS* genotype where females were twice as common as males. In fact, among all the female patients in our cohort (*N* = 57), the *2R/2R TYMS* genotype was present in 20 female patients (35%). Moreover, among patients with TYMS genotypes that predict decreased TS expression, *2R/2R TYMS* genotype was the most common *TYMS* genotype seen in female patients (57%). Of note, the three patients with *4R* containing *TYMS* genotypes (*2R/4R, 3R/4R, and 4R/3RG*) were all males. *TYMS* genotyping in patients with GI malignancies with different sex background is summarized in Table 2.

### Ethnic differences

Caucasians were the majority in both genotypes that predict decreased TS expression and in genotypes (3RG/3RG and 3RG/3RC) that predict increased TS expression. Among patients with TYMS genotypes that predict decreased TS expression, *2R/2R TYMS* genotype was the most common *TYMS* genotype seen in African American patients (60%). Of note, the three patients with *4R* containing *TYMS* genotypes (*2R/4R*, *3R/4R*, and *4R/3RG)* were all African Americans. There was minimal representation of other ethnic backgrounds in our cohort. *TYMS* genotyping in patients with GI malignancies with mixed racial background is summarized in Table 2.

### Adverse events

The frequency of grade 1–2 and grade 3–4 FrAEs in patients with *TYMS* genotypes that predict increased TS expression (*3RG/3RG*, *3RG/3RC*, *2R/3RG*, *2R/4R*, *3R/4R*, *4R/3RG*) and *TYMS* genotypes that predict decreased TS expression (*2R/2R*, *2R/3RC*, *3RC/3RC*) is summarized in Table 3. None of the patients have died as a consequence of fluoropyrimidine-induced toxicities. The most commonly experienced adverse event in both group of patients with grade 3–4 FrAEs was diarrhea. Among patients with *TYMS* genotypes that predict decreased TS expression (*2R/2R*, *2R/3RC*, *3RC/3RC*), the frequency of grade 1–2 and grade 3–4 FrAEs was further explored. Diarrhea was the most experienced grade 3–4 FrAE in patients with *2R/2R TYMS* genotype, while skin toxicity was the most experienced grade 3–4 FrAE in patients with *TYMS 2R/3RC*. Of note, grade 3–4 neutropenia and vasospasm were more experienced in patients with *TYMS 3RC/3RC* compared to *2R/2R* and *2R/3RC TYMS* genotypes. The frequency of grade 1–2 and grade 3–4 FrAEs in patients with *TYMS* genotypes that predict decreased TS expression (*2R/2R*, *2R/3RC*, *3RC/3RC*) is summarized in Table 4.

**Table 3:** The frequency of grade 1–2 and grade 3–4 fluoropyrimidines-related adverse events in patients with TYMS genotypes that predict increased and decreased TS expression.

Adverse events	TYMS genotypes that predict increased TS expression (*N* = 55)		TYMS genotypes that predict decreased TS expression (*N* = 71)	
	Grade 1–2	Grade 3–4	Grade 1–2	Grade 3–4
Hematological	*N* (%)	*N* (%)	*N* (%)	*N* (%)
Neutropenia	16 (29)	3 (5)	21 (30)	4 (6)
Anemia	10 (18)	0 (0)	16 (23)	0 (0)
Thrombocytopenia	4 (7)	0 (0)	6 (8)	0 (0)
Neutropenic fever		0 (0)	0 (0)	0 (0)
Non-hematological	*N* (%)	*N* (%)	*N* (%)	*N* (%)
Mucositis	4 (7)	3 (5)	4 (6)	3 (4)
Nausea	17 (31)	2 (4)	23 (32)	2 (3)
Vomiting	3 (5)	2 (4)	5 (7)	1 (1)
Diarrhea	4 (7)	9 (16)	7 (13)	11 (15)
Neurotoxicity	3 (5)	0 (0)	3 (4)	4 (6)
Skin toxicity	3 (5)	3 (5)	1 (1)	8 (11)
Fatigue	30 (55)	2 (4)	27 (38)	5 (7)
Vasospasm	0 (0)	1 (2)	0 (0)	4 (6)

**Table 4:** The frequency of grade 1–2 and grade 3–4 fluoropyrimidines-related adverse events in patients with *TYMS* genotypes that predict decreased TS expression *(2R/2R, 2R/3RC, and 3RC/3RC* genotypes).

Adverse events	TYMS genotypes that predict decreased TS expression (*N* = 71)					
	2R/2R (*N* = 31)		2R/3RC (*N* = 31)		3RC/3RC (*N* = 9)	
	G 1–2	G 3–4	G 1–2	G 3–4	G 1–2	G 3–4
Hematological	*N* (%)	*N* (%)	*N* (%)	*N* (%)	*N* (%)	*N* (%)
Neutropenia	11 (35)	1 (3)	9 (29)	1 (3)	1 (11)	2 (22)
Anemia	8 (26)	0 (0)	6 (19)	0 (0)	2 (22)	0 (0)
Thrombocytopenia	3 (10)	0 (0)	3 (10)	0 (0)	0 (0)	0 (0)
Neutropenic fever	0 (0)	0 (0)	0 (0)	0 (0)	0 (0)	0 (0)
Non-hematological	*N* (%)	*N* (%)	*N* (%)	*N* (%)	*N* (%)	*N* (%)
Mucositis	2 (6)	3 (10)	1 (3)	0 (0)	1 (11)	0 (0)
Nausea	13 (42)	2 (6)	9 (29)	0 (0)	1 (11)	0 (0)
Vomiting	2 (6)	1 (3)	2 (6)	0 (0)	1 (11)	0 (0)
Diarrhea	4 (13)	6 (19)	2 (6)	3 (10)	1 (11)	2 (22)
Neurotoxicity	0 (0)	2 (6)	3 (10)	2 (6)	0 (0)	0 (0)
Skin toxicity	1 (3)	3 (10)	0 (0)	4 (13)	0 (0)	1 (11)
Fatigue	17 (54)	2 (6)	8 (26)	3 (10)	2 (22)	0 (0)
Vasospasm	0 (0)	1 (3)	0 (0)	1 (3)	0 (0)	2 (22)

### Statistical analysis

Among patients with *TYMS* genotypes that predict increased TS expression (*N* = 55), 12 patients (22%) had grade 3–4 FrAEs, while among patients with *TYMS* genotypes that predict decreased TS expression, 30 patients (42%) had grade 3–4 FrAEs (*p* = 0.0219). Given the observed statistically significant difference, we explored the impact of the different *TYMS* genotypes that predict decreased TS expression (*2R/2R*, *2R/3RC*, *3RC/3RC*) on the observed results. Compared to patients with genotypes predicting increased TS expression, 17 out of 31 patients (55%) with 2R/2R TYMS genotype had grade 3–4 FrAEs (*p* = 0.0039), while only 15 out 40 patients (38%) with *2R/3RC* or *3RC/3RC TYMS* genotypes had grade 3–4 FrAEs (*p* = 0.1108). The association between grade 3–4 FrAEs and *TYMS* genotypes is shown in Table 5. Statistical analysis was performed using Fisher’s exact test.

**Table 5:** The association between grade 3–4 fluoropyrimidines-related adverse events (FrAEs) and TYMS genotypes.

Patients	Grade 3–4 FrAEs, *N* [%]	*p*
Patients with genotypes that predict increased TS expression (*N* = 55)	12 [22%]	*p* = 0.0219
Patients with genotypes that predict decreased TS expression (*N* = 71)	30 [42%]	
Patients with genotypes that predict increased TS expression (*N* = 55)	12 [22%]	*p* = 0.0039
Patients with 2*R*/2*R* genotype that predict decreased TS expression (*N* = 31)	17 [55%]	
Patients with genotypes that predict increased TS expression (*N* = 55)	12 [22%]	*p* = 0.1108
Patients with 2R/3RC and 3RC/3RC TYMS genotypes that predict decreased TS expression (*N* = 40)	15 [38%]	

Statistical analysis was performed using Fisher’s exact test.

## Discussion

The prevalence of 2R/2R *TYMS* genotype in different ethnic background is quite variable. In Caucasian Americans, the prevalence of 2R/2R *TYMS* genotype in infants with conotruncal heart defects and control group was 21% and 26%, respectively. In the same study, the prevalence of 2R/2R *TYMS* genotype in American Hispanics was 17% and 18%, respectively [27]. In children with acute lymphoblastic leukemia (ALL) and matched control, the prevalence of 2R/2R *TYMS* genotype, respectively, was 21% and 24% in the Netherlands, 21% and 21% in Germany, 25% and 24% in United kingdom, and 25% and 30% in Slovenia [28–31]. In patients with colorectal cancer, the prevalence of 2R/2R *TYMS* genotype was 18% in Hungary and 28% in Denmark [17, 32].

Many studies have explored the prevalence of 2R/2R *TYMS* genotype in Hispanic population as well. In healthy volunteers from Argentina, the reported prevalence is 26% [33]. In Brazil, the prevalence of 2R/2R *TYMS* genotype in children with ALL and matched control was 26% and 18%, respectively, in one study and 21% and 24%, respectively, in another study [34, 35]. In Mexico, the prevalence of 2R/2R *TYMS* genotype in patients with colorectal cancer and healthy subjects is 22% and 19%, respectively.

The prevalence of 2R/2R *TYMS* genotype in other ethnicities (Asians and Indians) showed lower reported prevalence compared to Caucasians and Hispanics. In children with ALL and matched control, the prevalence of 2R/2R *TYMS* genotype, respectively, was 16% and 0% in one study and 1% and 0% in another study from Indonesia. The prevalence was 2% and 2% in Singapore and 19% and 10% in India [36–39]. Table 6 summarizes several studies that explored the prevalence of 2R/2R *TYMS* genotype.

**Table 6:** Summary of several studies that explored the prevalence of 2R/2R *TYMS* genotype.

Author	Population	*N*	Prevalence *N* (%)
Zhu	Case and control infants (American Caucasians)	78 and 132	16 (21) and 34 (26)
Zhu	Case and control infants (American Hispanics)	144 and 396	25 (17) and 70 (18)
De Jonge	Children with ALL and matched control (Netherlands)	244 and 491	51 (21) and 116 (24)
Gast	Children with ALL and matched control (Germany)	457 and 541	95 (21) and 111 (21)
Lightfoot	Children with ALL and matched control (UK)	759 and 754	193 (25) and 181(24)
Petra	Children with ALL and matched control (Slovenia)	68 and 252	17 (25) and 76 (30)
Adleff	Colorectal (Hungary)	102	18 (17.6)
Kristensen	Colorectal (Denmark)	122	34 (28)
Vazquez	Healthy volunteers (Argentina)	199	43 (26.1)
Canalle	Children with ALL and matched control (Brazil)	126 and 300	33 (26) and 53 (18)
Silva	Children with ALL and matched control (Brazil)	140 and 390	25 (18) and 66 (17)
Gallegos-Arreola	Colorectal and healthy subjects (Mexico)	347 and 456	77 (22) and 85 (19)
Chan	Children with ALL and matched control (Indonesia)	184 and 177	30 (16) and 0 (0)
Giovannetti	Children with ALL and matched control (Indonesia)	71 and 44	1 (1) and 0 (0)
Yeoh	Children with ALL and matched control (Singapore)	518 and 652	12 (2) and 15 (2)
Nazki	Children with ALL and matched control (India)	43 and 144	8 (19) and 14 (10)

In our cohort of 126 patients, the prevalence of 2R/2R *TYMS* genotype was 24.6% of the total cohort and 44% of the patients with genotypes that predict decreased expression of TS. Among Caucasians (*N* = 80), 19 patients (24%) had 2R/2R *TYMS* genotype, which is not very different from the reported prevalence of 2R/2R *TYMS* genotype in Caucasians in America or Europe. Among African Americans (*N* = 43), 12 patients (28%) had 2R/2R *TYMS* genotype, which is slightly higher than the prevalence of 2R/2R *TYMS* genotype in Caucasians. The prevalence of 2R/2R *TYMS* genotype in African Americans is not well established. In one study that explored pharmacogenomics in patients with colorectal cancer, 36 patients were African Americans, and among this group, 25 patients (69%) had either 2R/2R or 2R/3R TYMS genotypes [40].

Among male patients (*N* = 69), 11 patients (16%) had 2R/2R *TYMS* genotype. Among female patients (*N* = 57), 20 patients (35%) had 2R/2R *TYMS* genotype. It is important to emphasize that 60% (12 patients) of the female patients in our cohort with 2R/2R *TYMS* genotype are African Americans. Several studies showed that women, especially African Americans, experienced more grade 3–4 fluoropyrimidine-associated toxicities compared to men. An underlying explanation is yet to be identified. The reported higher prevalence of 2R/2R *TYMS* genotype in female African American patients in our study might represent one possible explanation. Certainly, this should be considered hypothesis-generating observation.

The role of 2R/2R *TYMS* genotype in predicting severe FrAEs is controversial. The association between 2R/2R *TYMS* genotype and FrAEs has been demonstrated in many but not all studies. In the positive studies, the sensitivity and positive predictive value were of limited clinical benefit. Among unselected 200 patients treated with 5-FU, grade 3–4 FrAEs were experienced in 44 patients (22%). In this group of patients (*N* = 44), 13 patients had 2R/2R *TYMS* genotype (sensitivity 30%). Among all the patients with 2R/2R *TYMS* genotype (*N* = 25), 13 patients experienced grade 3–4 FrAEs (positive predictive value 52%) [7, 19, 22, 23].

On the other hand, several other studies failed to show a positive association between 2R/2R *TYMS* genotype and FrAEs [23–26]. In one prospective study where *TYMS* genotyping was used to select the chemotherapy of choice in patients with rectal cancer, the rate of grade 3–4 FrAEs was less in patients with *2R/2R*, *2R/3R*, or *2R/4R TYMS* genotypes compared to patients with *3R/3R* or *3R/4R TYMS* genotypes (30% vs 54%). Moreover, the hospitalization rate was lower at 16% vs 34% [26].

Our patients with 2R/2R *TYMS* genotype experienced different grade 3–4 hematological and non-hematological FrAEs. Diarrhea was the most common experienced grade 3–4 FrAEs. Other adverse events include neutropenia, mucositis, nausea, vomiting, neurotoxicity, skin toxicity, fatigue, and vasospasm. In our cohort, the association between 2R/2R *TYMS* genotype and FrAEs was noticeable. Compared to patients with genotypes predicting increased TS expression, *2R/2R* TYMS genotype was the only genotype among genotypes predicting decreased TS expression that had statistically significant association with grade 3–4 FrAEs (*p* = 0.0039). The association between the other genotypes (*2R/3RC* and *3RC/3RC TYMS* genotypes) and grade 3–4 FrAEs did not reach statistical significance (*p* = 0.1108).

Our study has several limitations. This study represents a single-institution experience with limited cohort of ethnic diversity. Our cohort was made of Caucasians and African Americans for the most part, and only three patients were from other ethnic backgrounds (Asian, Hispanic, and Indian American). Our cohort is also quite heterogenous regarding the primary site of the tumor and stage. It is also important to recognize that this study is a retrospective study and there are inherent limitations with a retrospective analysis, particularly regarding selection bias. *TYMS* genotyping strategies were quite variable as *TYMS* genotyping was at the discretion of the treating medical oncologist, and the selected treatment included several different fluoropyrimidine-based regimens. The medical oncologists followed the recommended dose management guidelines per package insert when they managed FrAEs. However, they still had a degree of variation in their practice. The process of attributing an experienced toxicity to 5-FU or capecitabine when they were part of fluoropyrimidine-based chemotherapy regimens was quite challenging sometimes. Every effort was made to make that attribution as accurate as possible. The aforementioned limitations should be kept in mind prior to drawing any conclusions.

## Conclusion

The prevalence of *TYMS 2R/2R* genotype in our cohort was 24.6%. Among Caucasians and African Americans, it was 24% and 28%, respectively. Polymorphism in the promoter region of *TYMS* gene that predict decreased TS expression due to *2R/2R* variant was associated with grade 3–4 FrAEs. These data suggest that genotyping patients who are not DPD deficient for TYMS might identify patients at risk of severe FrAEs.

The *2R/2R TYMS* genotype had a very unique sex and ethnic distribution. Among patients with TYMS genotypes that predict decreased TS expression (*2R/2R*, *2R/3RC*, *3RC/3RC*), *2R/2R TYMS* genotype was the most common *TYMS* genotype seen in female patients (57%) and in African American patients (60%). The reported higher prevalence of *2R/2R* *TYMS* genotype in female African American patients in our study might represent one possible explanation for why women, especially African Americans, experience more grade 3–4 FrAEs.

## References

[1] Hoff PM, Cassidy J, Schmoll HJ. The evolution of fluoropyrimidine therapy: from intravenous to oral. Oncologist. 2001;6:63–11. 10.1634/theoncologist.6-suppl_4-3 11585968

[2] Huang WY, Ho CL, Lee CC, Hsiao CW, Wu CC, Jao SW (2017). Oral tegafur-uracil as metronomic therapy following intravenous FOLFOX for stage III colon cancer. PLoS ONE.

[3] Cassidy J, Saltz L, Twelves C, Van Cutsem E, Hoff P, Kang Y (2011). Efficacy of capecitabine versus 5-fluorouracil in colorectal and gastric cancers: a meta-analysis of individual data from 6171 patients. Ann Oncol.

[4] Ershler WB (2006). Capecitabine monotherapy: safe and effective treatment for metastatic breast cancer. Oncologist..

[5] Vermorken JB, Remenar E, van Herpen C, Gorlia T, Mesia R, Degardin M (2007). Cisplatin, fluorouracil, and docetaxel in unresectable head and neck cancer. N Engl J Med.

[6] Twelves C, Wong A, Nowacki MP, Abt M, Burris H, Carrato A (2005). Capecitabine as adjuvant treatment for stage III colon cancer. N Engl J Med.

[7] Lecomte T, Ferraz JM, Zinzindohoue F, Loriot MA, Tregouet DA, Landi B (2004). Thymidylate synthase gene polymorphism predicts toxicity in colorectal cancer patients receiving 5-fluorouracil-based chemotherapy. Clin Cancer Res..

[8] Khushman M, Patel GK, Hosein PJ, Laurini JA, Cameron D, Clarkson DR (2018). Germline pharmacogenomics of DPYD*9A (c.85T>C) variant in patients with gastrointestinal malignancies treated with fluoropyrimidines. J Gastrointest Oncol.

[9] Thorn CF, Marsh S, Carrillo MW, McLeod HL, Klein TE, Altman RB (2011). PharmGKB summary: fluoropyrimidine pathways. Pharmacogenet Genomics.

[10] Lu Z, Zhang R, Carpenter JT, Diasio RB (1998). Decreased dihydropyrimidine dehydrogenase activity in a population of patients with breast cancer: implication for 5-fluorouracil-based chemotherapy. Clin Cancer Res..

[11] Etienne MC, Milano G, Renee N, Lagrange JL, Dassonville O, Thyss A (1995). [Population study of dihydropyrimidine dehydrogenase in cancer patients]. Bull Cancer..

[12] Mattison LK, Fourie J, Desmond RA, Modak A, Saif MW, Diasio RB (2006). Increased prevalence of dihydropyrimidine dehydrogenase deficiency in African-Americans compared with Caucasians. Clin Cancer Res.

[13] Offer SM, Fossum CC, Wegner NJ, Stuflesser AJ, Butterfield GL, Diasio RB (2014). Comparative functional analysis of DPYD variants of potential clinical relevance to dihydropyrimidine dehydrogenase activity. Cancer Res.

[14] Amstutz U, Henricks LM, Offer SM, Barbarino J, Schellens JHM, Swen JJ (2018). Clinical Pharmacogenetics Implementation Consortium (CPIC) guideline for dihydropyrimidine dehydrogenase genotype and fluoropyrimidine dosing: 2017 update. Clin Pharm Ther.

[15] Maharjan AS, McMillin GA, Patel GK, Awan S, Taylor WR, Pai S (2019). The prevalence of DPYD*9A(c.85T>C) genotype and the genotype-phenotype correlation in patients with gastrointestinal malignancies treated with fluoropyrimidines: updated analysis. Clin Colorectal Cancer.

[16] Parker WB, Cheng YC (1990). Metabolism and mechanism of action of 5-fluorouracil. Pharm Ther.

[17] Kristensen MH, Pedersen PL, Melsen GV, Ellehauge J, Mejer J (2010). Variants in the dihydropyrimidine dehydrogenase, methylenetetrahydrofolate reductase and thymidylate synthase genes predict early toxicity of 5-fluorouracil in colorectal cancer patients. J Int Med Res.

[18] Mandola MV, Stoehlmacher J, Muller-Weeks S, Cesarone G, Yu MC, Lenz HJ (2003). A novel single nucleotide polymorphism within the 5′ tandem repeat polymorphism of the thymidylate synthase gene abolishes USF-1 binding and alters transcriptional activity. Cancer Res.

[19] Pullarkat ST, Stoehlmacher J, Ghaderi V, Xiong YP, Ingles SA, Sherrod A (2001). Thymidylate synthase gene polymorphism determines response and toxicity of 5-FU chemotherapy. Pharmacogenomics J.

[20] Thomas F, Hoskins JM, Dvorak A, Tan BR, McLeod HL (2010). Detection of the G>C SNP and rare mutations in the 28-bp repeat of TYMS using gel-based capillary electrophoresis. Pharmacogenomics..

[21] Gallegos-Arreola MP, Zuniga-Gonzalez GM, Sanchez-Lopez JY, Cruz AYN, Peralta-Leal V, Figuera LE (2018). TYMS 2R3R polymorphism and DPYD [IVS]14+1G>A gene mutation in Mexican colorectal cancer patients. Acta Biochim Pol.

[22] Ichikawa W, Takahashi T, Suto K, Sasaki Y, Hirayama R (2006). Orotate phosphoribosyltransferase gene polymorphism predicts toxicity in patients treated with bolus 5-fluorouracil regimen. Clin Cancer Res.

[23] Rosmarin D, Palles C, Church D, Domingo E, Jones A, Johnstone E (2014). Genetic markers of toxicity from capecitabine and other fluorouracil-based regimens: investigation in the QUASAR2 study, systematic review, and meta-analysis. J Clin Oncol.

[24] Loganayagam A, Arenas Hernandez M, Corrigan A, Fairbanks L, Lewis CM, Harper P (2013). Pharmacogenetic variants in the DPYD, TYMS, CDA and MTHFR genes are clinically significant predictors of fluoropyrimidine toxicity. Br J Cancer.

[25] Sharma R, Hoskins JM, Rivory LP, Zucknick M, London R, Liddle C (2008). Thymidylate synthase and methylenetetrahydrofolate reductase gene polymorphisms and toxicity to capecitabine in advanced colorectal cancer patients. Clin Cancer Res.

[26] Tan BR, Thomas F, Myerson RJ, Zehnbauer B, Trinkaus K, Malyapa RS (2011). Thymidylate synthase genotype-directed neoadjuvant chemoradiation for patients with rectal adenocarcinoma. J Clin Oncol.

[27] Zhu H, Yang W, Shaw N, Perloff S, Carmichael SL, Finnell RH (2012). Thymidylate synthase polymorphisms and risk of conotruncal heart defects. Am J Med Genet. A.

[28] de Jonge R, Tissing WJ, Hooijberg JH, Jansen G, Kaspers GJ, Lindemans J (2009). Polymorphisms in folate-related genes and risk of pediatric acute lymphoblastic leukemia. Blood..

[29] Gast A, Bermejo JL, Flohr T, Stanulla M, Burwinkel B, Schrappe M (2007). Folate metabolic gene polymorphisms and childhood acute lymphoblastic leukemia: a case-control study. Leukemia..

[30] Lightfoot TJ, Johnston WT, Painter D, Simpson J, Roman E, Skibola CF (2010). Genetic variation in the folate metabolic pathway and risk of childhood leukemia. Blood..

[31] Petra BG, Janez J, Vita D (2007). Gene-gene interactions in the folate metabolic pathway influence the risk for acute lymphoblastic leukemia in children. Leuk Lymphoma..

[32] Adleff V, Hitre E, Koves I, Orosz Z, Hajnal A, Kralovanszky J (2004). Heterozygote deficiency in thymidylate synthase enhancer region polymorphism genotype distribution in Hungarian colorectal cancer patients. Int J Cancer.

[33] Vazquez C, Orlova M, Scibona P, Diaz Arce H, Pallotta MG, Belloso WH. Prevalence of thymidylate synthase gene 5′-untranslated region variants in an Argentinean sample. Genet Mol Res. 2017;16. 10.4238/gmr16019367 28128421

[34] Canalle R, Silveira VS, Scrideli CA, Queiroz RG, Lopes LF, Tone LG (2011). Impact of thymidylate synthase promoter and DNA repair gene polymorphisms on susceptibility to childhood acute lymphoblastic leukemia. Leuk Lymphoma.

[35] Silva RM, Fontes AC, Silva KA, Sant’Ana TA, Ramos FJ, Marques-Salles Tde J (2013). Polymorphisms involved in folate metabolism pathways and the risk of the development of childhood acute leukemia. Genet Test Mol Biomark.

[36] Chan JY, Ugrasena DG, Lum DW, Lu Y, Yeoh AE (2011). Xenobiotic and folate pathway gene polymorphisms and risk of childhood acute lymphoblastic leukaemia in Javanese children. Hematol Oncol.

[37] Giovannetti E, Ugrasena DG, Supriyadi E, Vroling L, Azzarello A, de Lange D (2008). Methylenetetrahydrofolate reductase (MTHFR) C677T and thymidylate synthase promoter (TSER) polymorphisms in Indonesian children with and without leukemia. Leuk Res.

[38] Yeoh AE, Lu Y, Chan JY, Chan YH, Ariffin H, Kham SK (2010). Genetic susceptibility to childhood acute lymphoblastic leukemia shows protection in Malay boys: results from the Malaysia-Singapore ALL Study Group. Leuk Res.

[39] Nazki FH, Masood A, Banday MA, Bhat A, Ganai BA (2012). Thymidylate synthase enhancer region polymorphism not related to susceptibility to acute lymphoblastic leukemia in the Kashmir population. Genet Mol Res.

[40] Sanoff HK, Sargent DJ, Green EM, McLeod HL, Goldberg RM (2009). Racial differences in advanced colorectal cancer outcomes and pharmacogenetics: a subgroup analysis of a large randomized clinical trial. J Clin Oncol.

